# Supplementary material for: SVA Regulation of Transposable Element Clustered Transcription within the Major Histocompatibility Complex Genomic Class II Region of the Parkinson’s Progression Markers Initiative
Source: Genes (Basel). 2024 Sep 9;15(9):1185. doi: 10.3390/genes15091185 (PMC11431313; doi:10.3390/genes15091185)
Supplement: Supplementary file 1 [file genes-15-01185-s001.zip › Figure S1.pdf]

Figure S1. Horizontal bar plots of SVA positive association (beta effect) (X-axis) with TE expression (Y-axis) in MHC class II genomic region, (a) NR_SVA_380 positive beta effect, (b) R_SVA_27 positive beta effect, (c) NR_SVA_381 positive beta effect, and (d) R_SVA_85 positive beta effect.

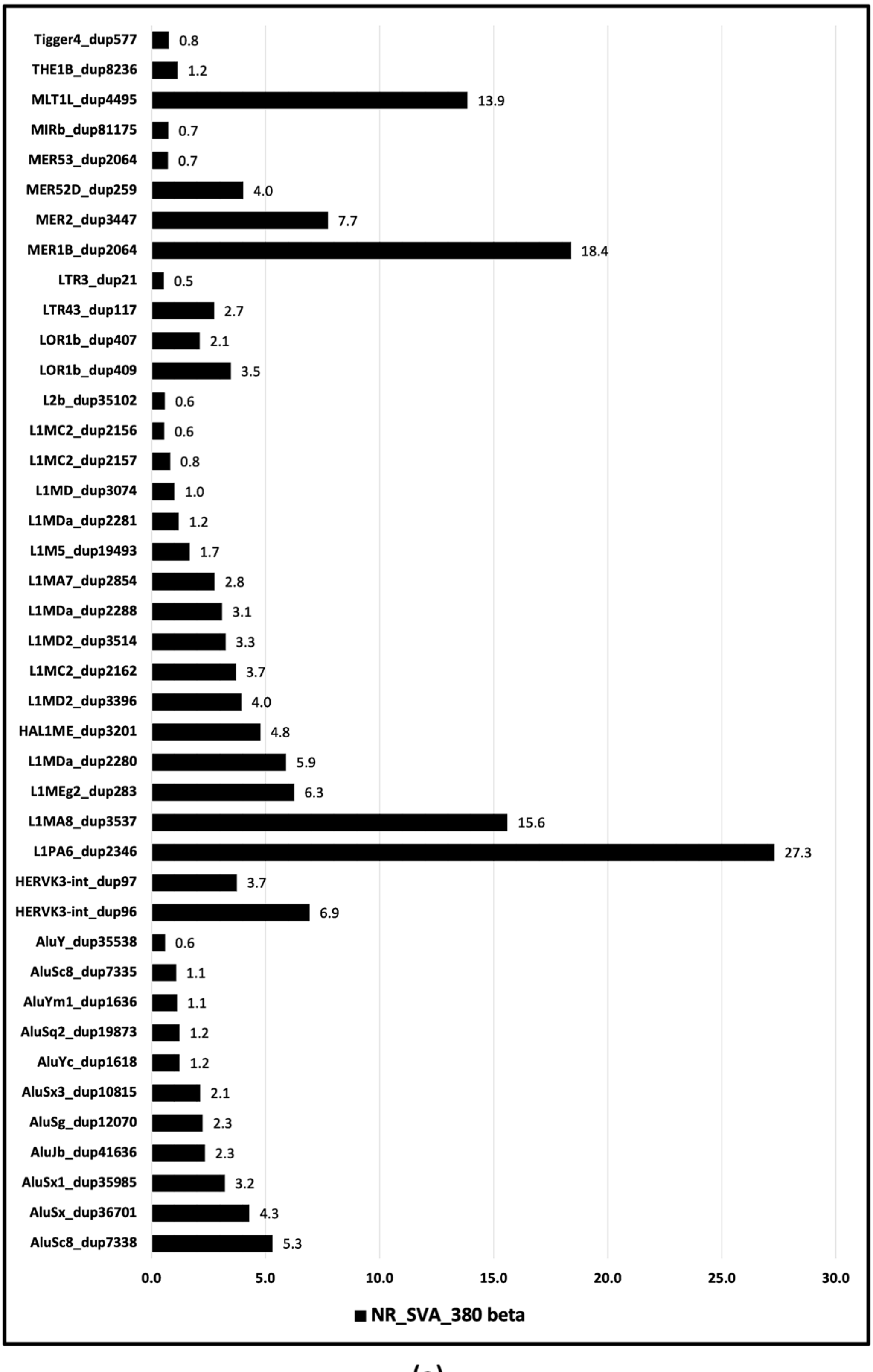

(a)

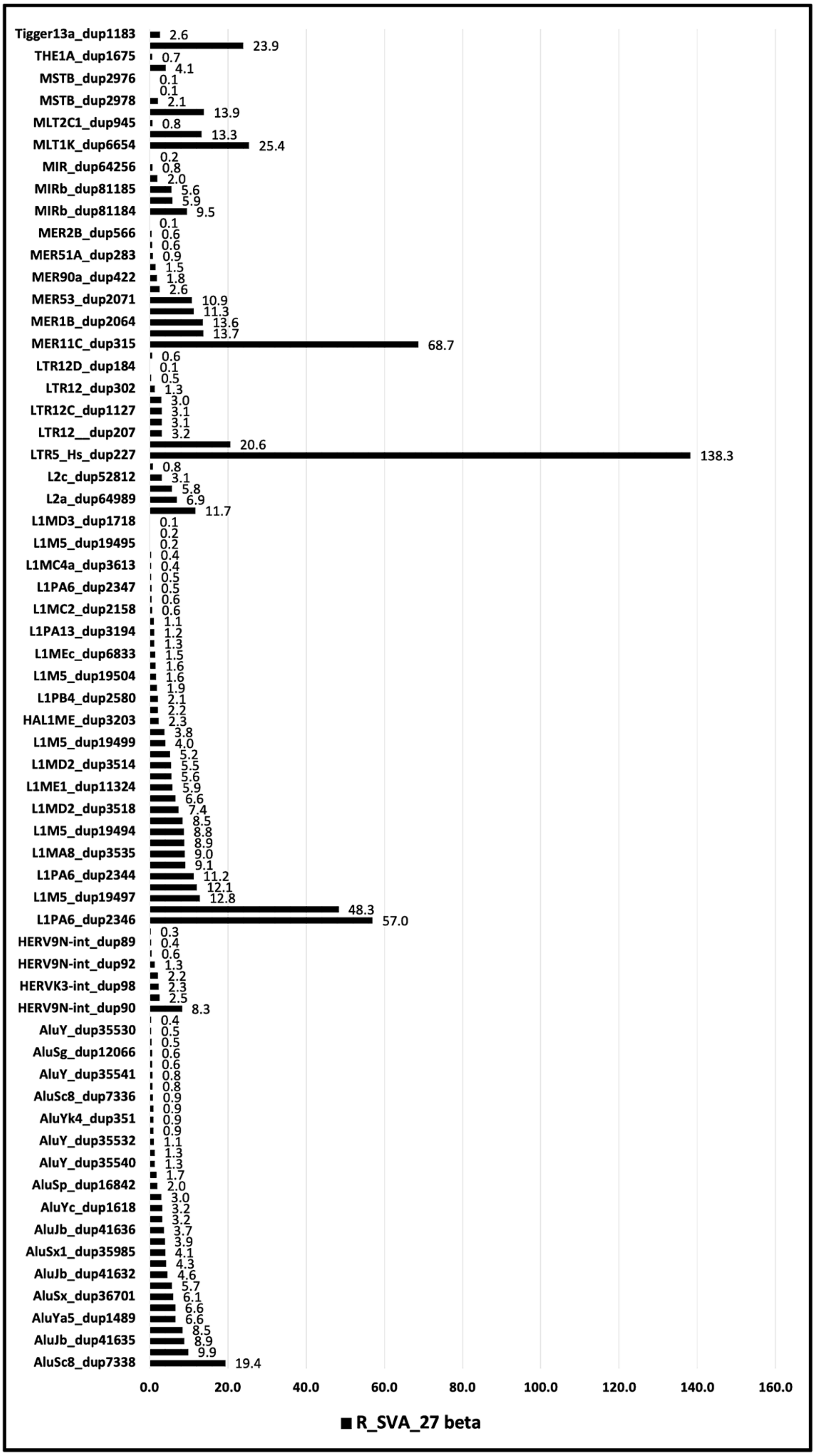
Tigger13a_dup1183
THE1A_dup1675
MSTB_dup2976
MSTB_dup2978
MLT2C1_dup945
MLT1K_dup6654
MIR_dup64256
MIRb_dup81185
MIRb_dup81184
MER2B_dup566
MER51A_dup283
MER90a_dup422
MER53_dup2071
MER1B_dup2064
MER11C_dup315
LTR12D_dup184
LTR12_dup302
LTR12C_dup1127
LTR12__dup207
LTR5_Hs_dup227
L2c_dup52812
L2a_dup64989
L1MD3_dup1718
L1M5_dup19495
L1MC4a_dup3613
L1PA6_dup2347
L1MC2_dup2158
L1PA13_dup3194
L1MEc_dup6833
L1M5_dup19504
L1PB4_dup2580
HAL1ME_dup3203
L1M5_dup19499
L1MD2_dup3514
L1ME1_dup11324
L1MD2_dup3518
L1M5_dup19494
L1MA8_dup3535
L1PA6_dup2344
L1M5_dup19497
L1PA6_dup2346
HERV9N-int_dup89
HERV9N-int_dup92
HERVK3-int_dup98
HERV9N-int_dup90
AluY_dup35530
AluSg_dup12066
AluY_dup35541
AluSc8_dup7336
AluYk4_dup351
AluY_dup35532
AluY_dup35540
AluSp_dup16842
AluYc_dup1618
AluJb_dup41636
AluSx1_dup35985
AluJb_dup41632
AluSx_dup36701
AluYa5_dup1489
AluJb_dup41635
AluSc8_dup7338
2.6
23.9
0.7
4.1
0.1
0.1
2.1
13.9
0.8
13.3
25.4
0.2
0.8
2.0
5.6
5.9
9.5
0.1
0.6
0.6
0.9
1.5
1.8
2.6
10.9
11.3
13.6
13.7
68.7
0.6
0.1
0.5
1.3
3.0
3.1
3.1
3.2
20.6
138.3
0.8
3.1
5.8
6.9
11.7
0.1
0.2
0.2
0.4
0.4
0.5
0.5
0.6
0.6
1.1
1.2
1.3
1.5
1.6
1.6
1.9
2.1
2.2
2.3
3.8
4.0
5.2
5.5
5.6
5.9
6.6
7.4
8.5
8.8
8.9
9.0
9.1
11.2
12.1
12.8
48.3
57.0
0.3
0.4
0.6
1.3
2.2
2.3
2.5
8.3
0.4
0.5
0.5
0.6
0.6
0.8
0.8
0.9
0.9
0.9
0.9
1.1
1.3
1.3
1.7
2.0
3.0
3.2
3.2
3.7
3.9
4.1
4.3
4.6
5.7
6.1
6.6
6.6
8.5
8.9
9.9
19.4
0.0
20.0
40.0
60.0
80.0
100.0
120.0
140.0
160.0
R_SVA_27 beta

(b)

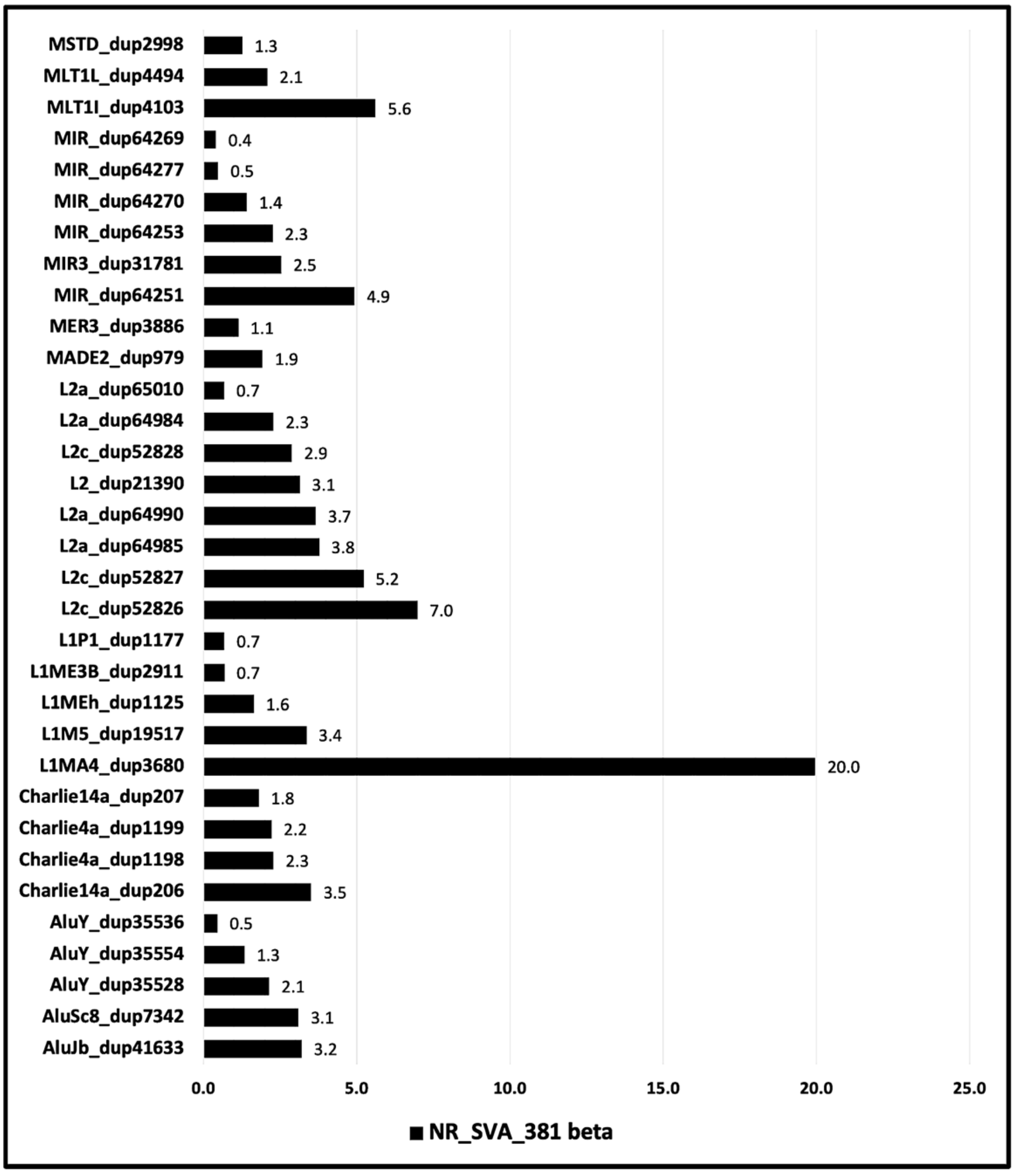
MSTD_dup2998 1.3
MLT1L_dup4494 2.1
MLT1I_dup4103 5.6
MIR_dup64269 0.4
MIR_dup64277 0.5
MIR_dup64270 1.4
MIR_dup64253 2.3
MIR3_dup31781 2.5
MIR_dup64251 4.9
MER3_dup3886 1.1
MADE2_dup979 1.9
L2a_dup65010 0.7
L2a_dup64984 2.3
L2c_dup52828 2.9
L2_dup21390 3.1
L2a_dup64990 3.7
L2a_dup64985 3.8
L2c_dup52827 5.2
L2c_dup52826 7.0
L1P1_dup1177 0.7
L1ME3B_dup2911 0.7
L1MEh_dup1125 1.6
L1M5_dup19517 3.4
L1MA4_dup3680 20.0
Charlie14a_dup207 1.8
Charlie4a_dup1199 2.2
Charlie4a_dup1198 2.3
Charlie14a_dup206 3.5
AluY_dup35536 0.5
AluY_dup35554 1.3
AluY_dup35528 2.1
AluSc8_dup7342 3.1
AluJb_dup41633 3.2
0.0 5.0 10.0 15.0 20.0 25.0
NR_SVA_381 beta

(c)

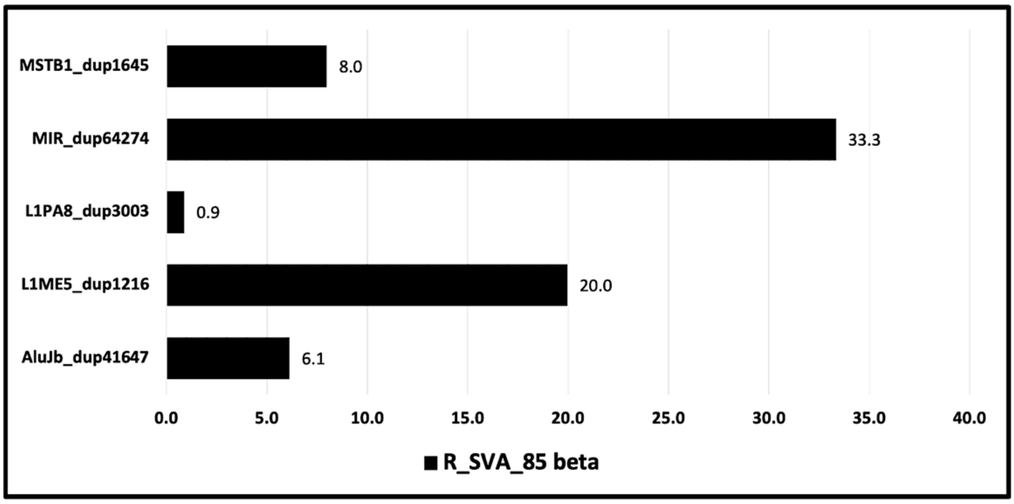
MSTB1_dup1645 8.0
MIR_dup64274 33.3
L1PA8_dup3003 0.9
L1ME5_dup1216 20.0
AluJb_dup41647 6.1
0.0 5.0 10.0 15.0 20.0 25.0 30.0 35.0 40.0
R_SVA_85 beta

(d)
