# Supplementary material for: SVA Regulation of Transposable Element Clustered Transcription within the Major Histocompatibility Complex Genomic Class II Region of the Parkinson’s Progression Markers Initiative
Source: Genes (Basel). 2024 Sep 9;15(9):1185. doi: 10.3390/genes15091185 (PMC11431313; doi:10.3390/genes15091185)
Supplement: Supplementary file 1 [file genes-15-01185-s001.zip › Figure S2.pdf]

**Figure S2. Five supplementary figures 2a to 2e of the associations between ENCODE cCREs and DNase hypersensitivity marks and expressed TE (eTE) clusters in the HLA class II genomic region.**

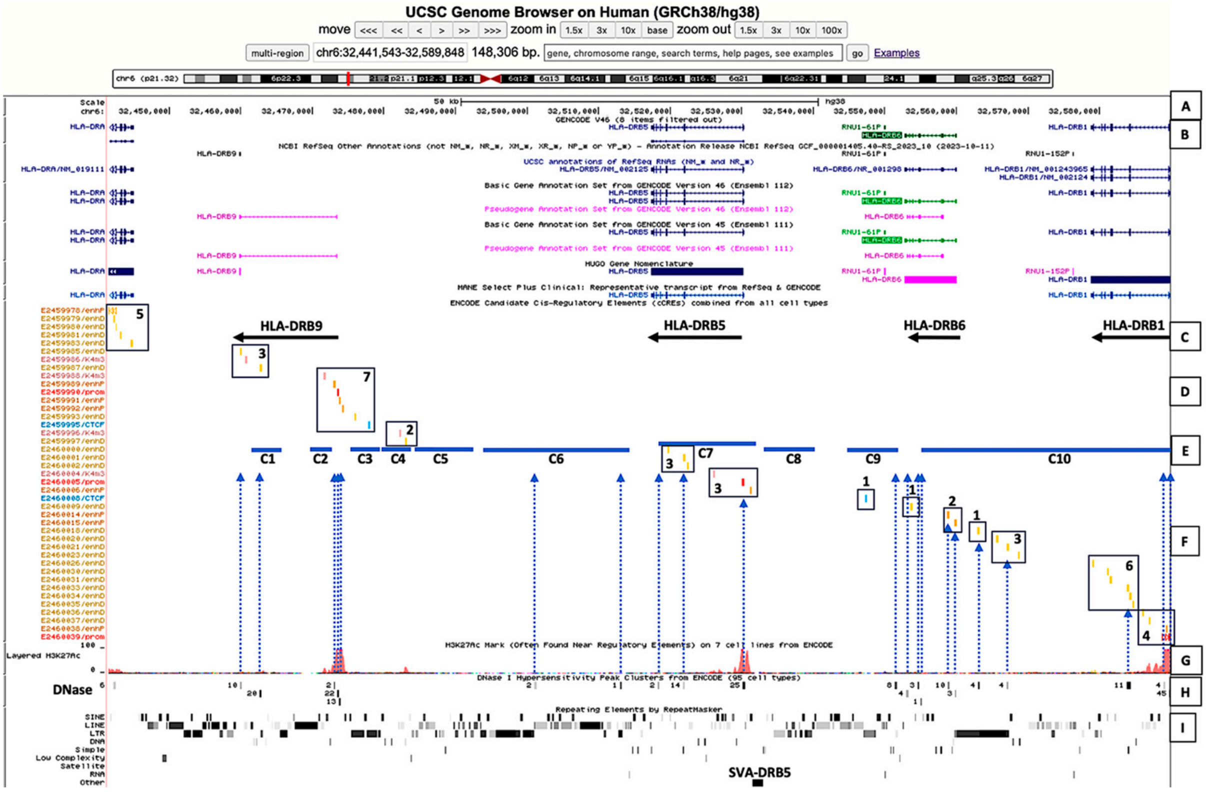


Figure S2a.

Cluster loci C1 to C10 of eTEs relative to gene loci HLA-DRB9, -DRB5, -DRB6, -DRB1; and 41 CRE (vertical coloured bars, labelled number of CREs in each box), 21 Dnase I hypersensitivity peak clusters (vertical dotted arrows), and H3K27Ac mark (red peaks) from ENCODE using UCSC browser. Boxed A to I listed vertically on the right-sided border indicate rows of interest: (A) chromosomal genomic position (bp), (B) gene positions, (C) gene positions redrawn, (D) various CREs (enhP, enhD, K4me3, prom, CTCF) as coloured vertical bars in boxes with indicated numbers of CREs in each box, (E) location of eTE clusters C1 to C10 indicated by horizontal blue lines, (F) CRE (continued from [D]) and Dnase I hypersensitivity peak loci (vertical dotted arrows), (G) H3K27Ac mark, (H) Dnase I hypersensitivity peak (same loci as indicated by vertical arrows in [F]), (I) Repeat elements identified by RepeatMasker.

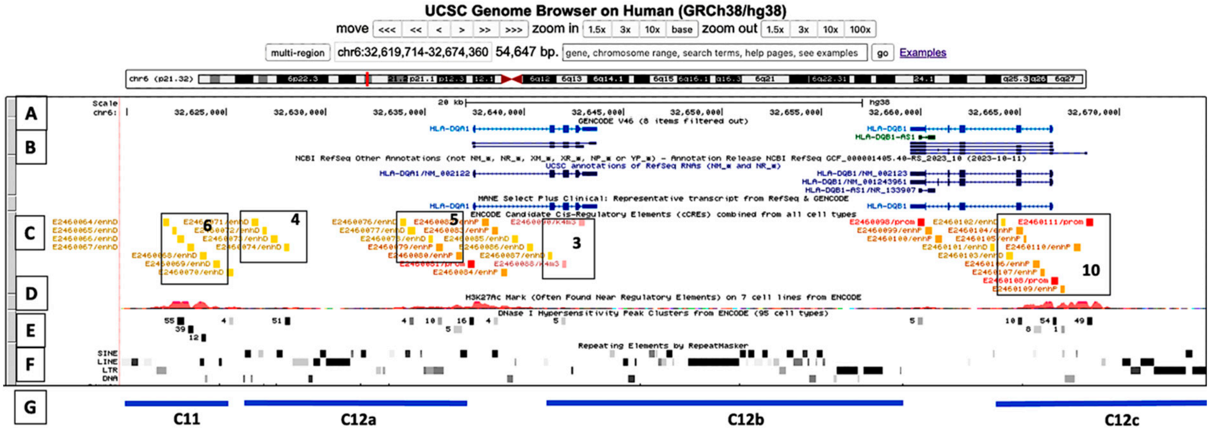


Figure S2b.
Cluster loci C11 and C12 of eTEs in row [G] relative to gene loci HLA-DQA1, and –DQB1 [row B], 28 overlapping CREs (vertical coloured bars, labelled number of CREs in each box in row [C]), 14 overlapping Dnase I hypersensitivity peak clusters (small numbered black bars in row [E)], H3K27Ac mark (red peaks) from ENCODE in row [D], repeat elements SINE, LINE, LTR and DNA in row [F] using UCSC browser outputs. Boxed A to G listed vertically on the left-sided border indicate rows of interest: (A) chromosomal genomic position (bp), (B) gene positions, (C) various CREs (enhP, enhD, K4me3, prom, CTCF) as coloured vertical bars in boxes with labelled numbers of CREs in each box, (D) H3K27Ac mark, (E) Dnase I hypersensitivity peak loci (small numbered bars), (F) repeat elements identified by RepeatMasker, and (G) cluster loci C11 and C12 (a, b and c) of eTEs reported in the present study.

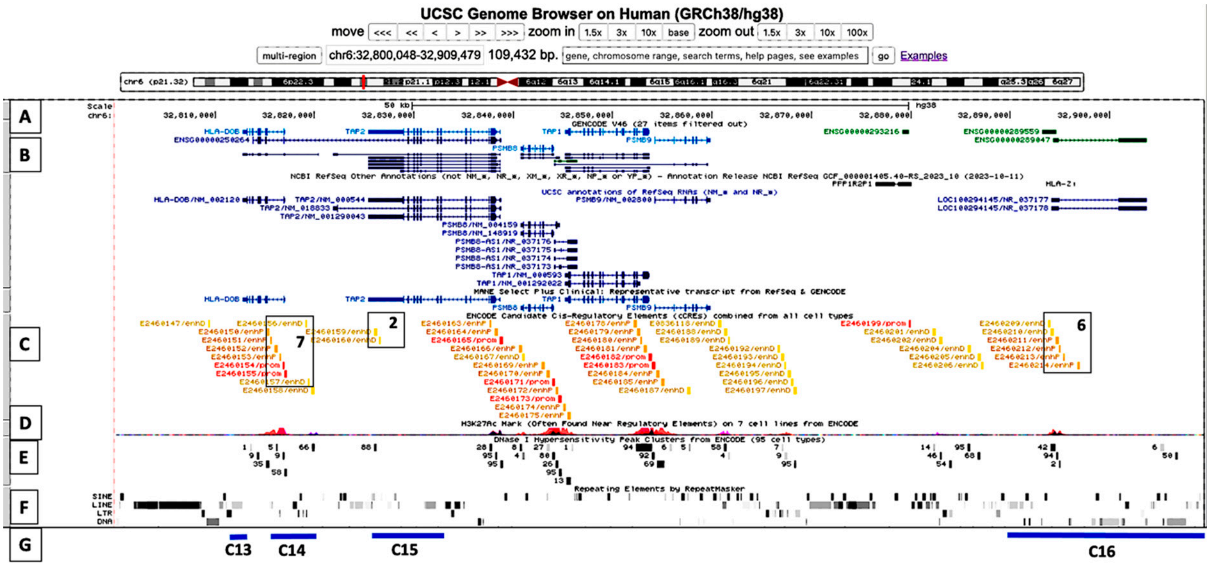


Figure S2c.
Cluster loci C13 and C16 of eTEs in row [G] relative to gene loci HLA-DOB, TAP2, and HLA-Z [row B], 15 overlapping CREs (vertical coloured bars, labelled number of CREs in each box in row ]C]), 11 overlapping Dnase I hypersensitivity peak clusters (small numbered black bars in row [E], H3K27Ac mark (red peaks) from ENCODE in row [D], repeat elements SINE, LINE, LTR and DNA in row [F] using UCSC browser outputs. Boxed A to G listed vertically on the left-sided border indicate rows of interest: (A) chromosomal genomic position (bp), (B) gene positions, (C) various CREs (enhP, enhD, K4me3, prom, CTCF) as coloured vertical bars in boxes with labelled numbers of CREs in each box, (D) H3K27Ac mark, (E) Dnase I hypersensitivity peak loci (small numbered bars), (F) repeat elements identified by RepeatMasker, and (G) cluster loci C13 to C16 of eTEs reported in the present study.

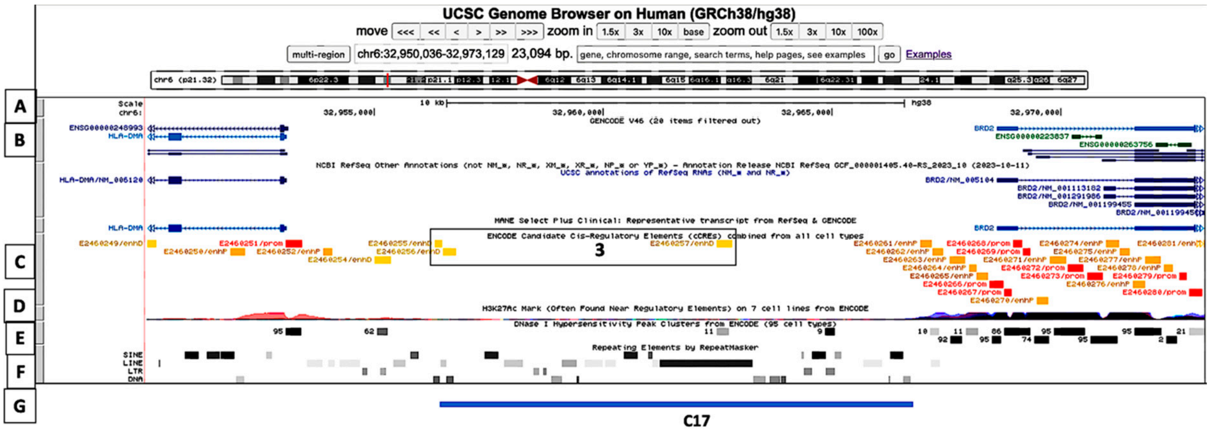


Figure S2d.
Cluster locus C17 of eTEs in row [G] relative to gene loci HLA-DMA and BRD2 [row B], 3 overlapping CREs (vertical coloured bars, labelled number of CREs in each box in row [C]), 2 overlapping Dnase I hypersensitivity peak clusters (numbered small black bars in row [E)], H3K27Ac mark (red, blue and black peaks) from ENCODE in row [D], repeat elements SINE, LINE, LTR and DNA in row [F] using UCSC browser outputs. Boxed A to G listed vertically on the left-sided border indicate rows of interest: (A) chromosomal genomic position (bp), (B) gene positions, (C) various CREs (enhP, enhD, K4me3, prom, CTCF) as coloured vertical bars in boxes with labelled numbers of CREs in each box, (D) H3K27Ac mark, (E) Dnase I hypersensitivity peak loci (small numbered bars), (F) repeat elements identified by RepeatMasker, and (G) cluster locus C17 of eTEs reported in the present study.

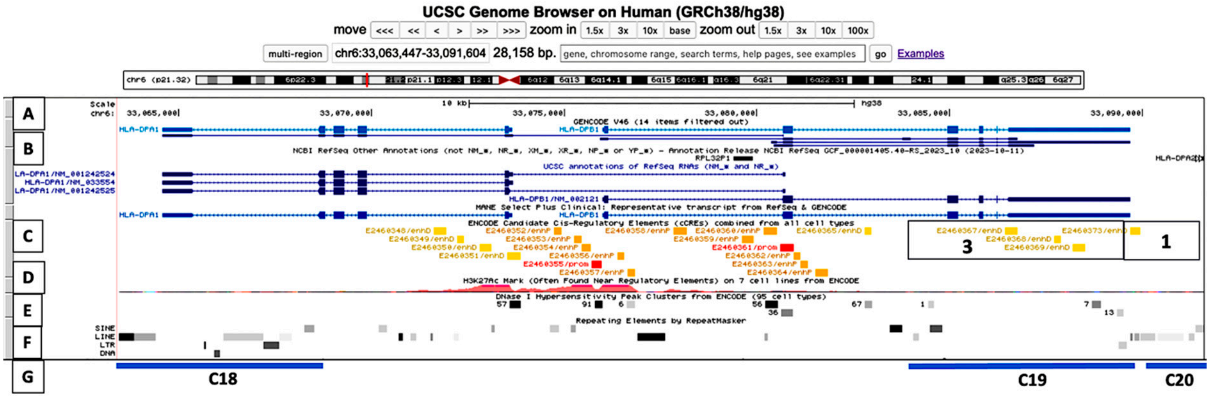


Figure S2e.
Cluster loci C18 and C20 of eTEs in row [G] relative to gene loci HLA-DPA1, -DPB1, -DPA2 [row B], 4 overlapping CREs (vertical coloured bars, labelled number of CREs in each box in row [C]), 3 overlapping Dnase I hypersensitivity peak clusters (numbered black bars in row [E)], H3K27Ac mark (red peaks) from ENCODE in row [D], repeat elements SINE, LINE, LTR and DNA in row [F] using UCSC browser outputs. Boxed A to G listed vertically on the left-sided border indicate rows of interest: (A) chromosomal genomic position (bp), (B) gene positions, (C) various CREs (enhP, enhD, K4me3, prom, CTCF) as coloured vertical bars in boxes with labelled numbers of CREs in each box, (D) H3K27Ac mark, (E) DNase I hypersensitivity peak loci (small numbered bars), (F) repeat elements identified by RepeatMasker, and (G) cluster loci C18 to C20 of eTEs reported in the present study.
